# Contralateral Inhibition of Click- and Chirp-Evoked Human Compound Action Potentials

**DOI:** 10.3389/fnins.2017.00189

**Published:** 2017-04-04

**Authors:** Spencer B. Smith, Jeffery T. Lichtenhan, Barbara K. Cone

**Affiliations:** ^1^Department of Speech, Language, and Hearing Sciences, University of ArizonaTucson, AZ, USA; ^2^Department of Otolaryngology, Washington University School of MedicineSt. Louis, MO, USA

**Keywords:** medial olivocochlear reflex, efferent auditory system, electrocochleography, compound action potential, chirps

## Abstract

Cochlear outer hair cells (OHC) receive direct efferent feedback from the caudal auditory brainstem via the medial olivocochlear (MOC) bundle. This circuit provides the neural substrate for the MOC reflex, which inhibits cochlear amplifier gain and is believed to play a role in listening in noise and protection from acoustic overexposure. The human MOC reflex has been studied extensively using otoacoustic emissions (OAE) paradigms; however, these measurements are insensitive to subsequent “downstream” efferent effects on the neural ensembles that mediate hearing. In this experiment, click- and chirp-evoked auditory nerve compound action potential (CAP) amplitudes were measured electrocochleographically from the human eardrum without and with MOC reflex activation elicited by contralateral broadband noise. We hypothesized that the chirp would be a more optimal stimulus for measuring neural MOC effects because it synchronizes excitation along the entire length of the basilar membrane and thus evokes a more robust CAP than a click at low to moderate stimulus levels. Chirps produced larger CAPs than clicks at all stimulus intensities (50–80 dB ppeSPL). MOC reflex inhibition of CAPs was larger for chirps than clicks at low stimulus levels when quantified both in terms of amplitude reduction and effective attenuation. Effective attenuation was larger for chirp- and click-evoked CAPs than for click-evoked OAEs measured from the same subjects. Our results suggest that the chirp is an optimal stimulus for evoking CAPs at low stimulus intensities and for assessing MOC reflex effects on the auditory nerve. Further, our work supports previous findings that MOC reflex effects at the level of the auditory nerve are underestimated by measures of OAE inhibition.

## Introduction

Cochlear outer hair cells (OHC) receive direct efferent feedback from the caudal auditory brainstem via the medial olivocochlear (MOC) nerve bundle. The MOC bundle inhibits OHC motility and indirectly modulates basilar membrane motion and inner hair cell (IHC) sensitivity—an effect termed the MOC reflex (Mountain, 1980; Siegel and Kim, 1982; Murugasu and Russell, 1996; Cooper and Guinan, 2003, 2006). Experiments in animal models have revealed that excitation of the MOC reflex “unmasks” signal representation in the auditory nerve by reducing mechano-electrical transduction of noise within the cochlea and therefore may play an active role in hearing in noise (Kawase and Liberman, 1993; Kawase et al., 1993). The functional importance of the MOC reflex in human hearing, however, remains unclear.

Because otoacoustic emissions (OAEs) likely originate from mechanics associated with OHC motility (Liberman et al., 2002; Cheatham et al., 2004; Dallos et al., 2008), they are sensitive to MOC reflex-induced changes in OHC function and provide a non-invasive, albeit indirect, method to study efferent effects in humans. In the classic contralateral inhibition of OAEs paradigm, OAEs are measured without and with presentation of a contralateral acoustic stimulus (CAS; e.g., broadband noise, BBN), which activates the uncrossed MOC fibers of the reflex circuit. Magnitude and/or phase differences between OAEs recorded without and with CAS are then used to quantify MOC reflex-induced shifts in OHC function (Guinan, 2006). Such studies have quantified characteristics of human MOC reflex strength (e.g., Backus and Guinan, 2007; Marshall et al., 2014), tuning (e.g., Veuillet et al., 1991; Chéry-Croze et al., 1993; Lilaonitkul and Guinan, 2009; Zhao and Dhar, 2012), and laterality (e.g., Francis and Guinan, 2010; Garinis et al., 2011). However, OAEs are *pre-neural* measurements and are therefore less informative about the “downstream” MOC effects on IHC excitation and the subsequent neural ensembles that mediate hearing.

Few experiments have reported MOC reflex effects on evoked compound action potentials (CAPs) from the human auditory nerve (Folsom and Owsley, 1987; Kawase and Takasaka, 1995; Chabert et al., 2002; Lichtenhan et al., 2016; Najem et al., 2016). Both the dearth of research in this area and the wide range of reported inhibition with CAS (2–20 dB) may stem from technical issues related to CAP inhibition measurements. For example, OAE experiments have shown that the effect of MOC reflex inhibition on OHC activity is more potent at lower stimulus levels (e.g., Hood et al., 1996); however, clicks and tone bursts presented at these levels evoke less synchronized neural responses from a smaller population of auditory nerve fibers and therefore produce CAP waveforms with poorer morphology than higher stimulus levels. Without adequate response averaging, CAP waveforms evoked by low- to moderate-level clicks or tone bursts are highly variable with poor signal-to-noise ratios and “true” physiologic changes attributable to the MOC reflex (i.e., reduction in CAP amplitude) are difficult to separate from measurement variation.

Stimuli evoking more robust CAP responses than clicks or tone bursts, such as rising frequency chirps, may circumvent some of the technical issues related to neural MOC reflex measurements. Unlike a click, which initiates synchronized responses predominately from more basal auditory nerve fibers (Kiang, 1975; Abdala and Folsom, 1995), chirps synchronize auditory nerve fiber excitation along the length of the cochlear spiral by correcting for temporal delays associated with tonotopicity (Shore and Nuttall, 1985; Fobel and Dau, 2004). Recently, Chertoff et al. (2010) demonstrated that chirps optimized for eliciting human CAPs produced significantly larger amplitudes than those evoked by clicks in young, normal-hearing adults at moderate to high stimulus levels (75–125 pSPL). The improved signal-to-noise ratio of chirp-evoked CAPs, compared to those from clicks, may thus provide a higher fidelity response to assay CAS-induced MOC reflex effects on the auditory nerve. Additionally, MOC fibers innervate the length of the cochlear spiral with tuning similar to afferent auditory nerve fibers (Warr, 1992). Chirp-evoked CAPs may therefore be more sensitive to the summed CAS-induced MOC reflex effects along the entire length of the cochlea and thus show greater inhibition than click-evoked CAPs.

In this experiment, we tested two hypotheses: (1) That chirps evoke larger CAP amplitudes than clicks using low to moderate stimulus levels, which engage the cochlear amplifier and are thus more sensitive to MOC effects and (2) That MOC reflex inhibition of chirp-evoked CAPs is larger than for click-evoked CAPs due to the broader basilar membrane area represented in chirp responses. To relate our findings to more commonly used MOC reflex assays, we also compared average chirp- and click-evoked CAP inhibition to click evoked OAE (CEOAE) inhibition measured in the same subjects.

## Methods

### Participants

The University of Arizona Human Subjects Protection Program approved the following methods which were carried out with written, informed consent from all subjects. Eighteen adult participants without history of neurologic or otologic disease were enrolled in the study; however, due to attrition, 14 subjects (average age 22.25 years; 10 females) completed all six testing sessions. Otoscopy examinations found that all ear canals were free of excess cerumen and that tympanic membranes (TMs) appeared healthy in all subjects. Participants had normal tympanograms bilaterally, defined as ear canal volume of 0.6–1.5 cc and peak-compensated static admittance between 0.3 and 1.4 mL (Margolis and Heller, 1987), and contralateral acoustic reflex thresholds to 1–10 kHz BBN ≥70 dB SPL, measured using conventional admittance methods (Sun, 2008). The latter requirement was to mitigate the possible involvement of middle ear muscle contractions during MOC inhibition measurements, although others have shown that acoustic reflex thresholds can be lower when measured using more sensitive techniques (e.g., Zhao and Dhar, 2010; Lichtenhan et al., 2016). Air conduction hearing thresholds from 0.25 to 8 kHz were within normal limits (≤25 dB HL) bilaterally for all subjects.

### Equipment and procedures

#### Stimulus generation and calibration

A 100-μs click and 10-ms chirp were used to evoke CAPs. The click was created using the Intelligent Hearing Systems Smart-EP stimulus generator (Intelligent Hearing Systems, Miami, FL). The chirp was created in WAV file format in MATLAB (The Mathworks, Inc., Natick, MA, USA) using a modified “O-Chirp” from Fobel and Dau (2004), as implemented by Chertoff et al. (2010). The O-Chirp is a flat-spectrum stimulus relating frequency to basilar membrane delay using parameters from stimulus frequency OAEs. To optimize the O-Chirp for evoking CAPs, forward traveling wave delays were estimated from Eggermont's (1979) derived-band CAP latencies as opposed to stimulus frequency OAEs. The relationship between basilar membrane delay in milliseconds and frequency was expressed as:

$$\begin{array}{l} \tau_{\text{BM}}={\text{c}}^{*}f^{\alpha } \end{array}$$

where 0.45 kHz ≤ *f* ≤ 10 kHz and c (0.69) and α (−77) are constants. The chirp WAV file was converted into a stimulus file suitable for presentation by the Intelligent Hearing Systems Smart-EP program.

The click and chirp were presented through ER-3A insert earphones (Etymotic Research, Elk Grove Village, IL) to a 2-cc coupler and calibrated in units of *dB peak-to-peak equivalent sound pressure level* (ppeSPL) using a 1,000 Hz tone as a reference (Burkard, 2006). Click and chirp spectra were comparable with the exception that the chirp had 3–5 dB less energy below ~3.5–4 kHz (see Chertoff et al., 2010, Figure 1).

**Figure 1:**
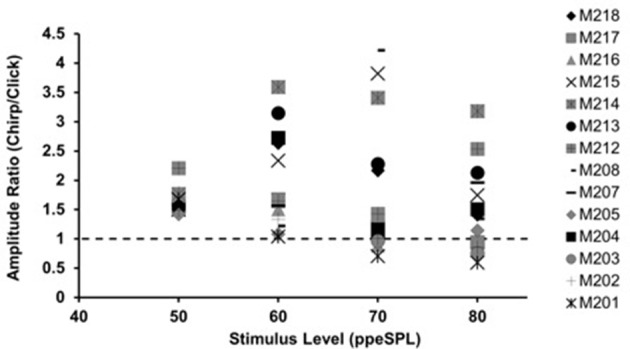
**Chirp-to-click CAP amplitude ratios for waveforms obtained without CAS**. Each symbol represents a single subject. Symbols falling above the dotted line indicate larger chirp responses than clicks.

Behavioral thresholds for clicks and chirps were obtained from the right ears of 18 subjects using a modified Hughson-Westlake procedure. Stimuli were presented at a starting presentation level of 50 dB ppeSPL. Presentation level was decreased by 4 dB after every positive response and increased by 2 dB after each failure to respond. Threshold was defined as the lowest presentation level at which three positive responses occurred. These measurements were made without electrodes in the ear canal, as our previous work demonstrated that TM electrode contact with the eardrum can influence audiometric thresholds, particularly to low frequencies (Smith et al., 2016). Average behavioral thresholds were 32 dB ppeSPL and 30 dB ppeSPL for clicks and chirps, respectively. While we express stimulus levels in units of dB ppeSPL throughout this paper, behavioral thresholds to clicks and chirps can be subtracted from these values to convert from dB ppeSPL to normalized hearing level (nHL).

#### Tympanic membrane electrodes

Using a modified protocol by Ferraro and Durrant (2006), we assembled TM electrodes in our laboratory that were suitable for our evoked potentials recording system. The electrodes were constructed from 11.43-cm long sections of PFA-insulated silver wire (0.1 mm gauge) encased in 10.16-cm long pieces of flexible silastic medical tubing. The PFA-insulation was removed from the last 0.635 cm of each end of the wire. One uninsulated end was crimped with a female machine pin that was connected to an electrode cable interfacing with the bio-amplifier. The other uninsulated end was bent to form a hook around a 0.25 gram wisp of cotton, and the end of the hook was tucked back into the opening of the silastic medical tubing to ensure that it did not directly make contact with the eardrum when it was inserted. Prior to each recording session, the cotton-tipped end of a TM electrode was saturated with 1-cc of Synapse electrode cream (Kustomer Kinetics, Arcadia, CA) using a 27-gauge needle. TM electrodes were inserted into the right ear canal of each subject and advanced until the TM was contacted, which was verified by subject report of the occlusion effect and by monitoring electrode impedance changes until they were consistently ≤7 kΩ on the Intelligent Hearing Systems bio-amplifier (Ferraro, 2010). Further confirmation of electrode contact with the TM was indicated by areas of acute redness and accumulation of electrode gel observed otoscopically after TM electrodes were removed at the end of each testing session (see Smith et al., 2016, Figure 1). Each electrode was held in place throughout the session by a 13 mm ER3-14A foam ear tip coupled to the ER-3A insert earphone.

#### CAP measurements and amplitude calculations

Each subject participated in six 2-h CAP recording sessions—three in which clicks were used to evoke CAPs and three in which chirps were used. The order in which subjects participated in click or chirp sessions was randomized. In every session, subjects were comfortably reclined in a lounge chair in an electromagnetically shielded sound booth and remained awake and alert throughout recordings. CAPs were acquired using a single-channel electrode montage: right TM electrode (+), left earlobe (−), and forehead ($\underline{\underline{⊥}}$). Waveforms were sampled at a rate of 40 kHz over a 25.6 ms epoch, filtered from 0.1 to 3 kHz, and amplified by 150,000. Stimulus presentation rate was Gaussian distributed from 9.1/s to 13.1/s with a mean rate of 11.1/s. This relatively slow range of presentation rates was selected to ensure that the *stimuli* did not temporally summate to activate the MOC reflex, which has been shown to affect OAE measurements at stimulus presentation rates as low as 30/s–50/s (Veuillet et al., 1991; Francis and Guinan, 2010; Boothalingam and Purcell, 2015). A Gaussian-distributed (i.e., “temporally jittered”) presentation rate was selected to facilitate subject alertness, as this may influence MOC reflex strength (Aedo et al., 2015).

CAP level-series measurements without and with CAS (1–10 kHz flat spectrum BBN at 60 dB SPL, delivered to left ears through an ER-2 earphone) were interleaved throughout the duration of each 2-h session with the exception that the first 20 min of the sixth session was devoted to CEOAE measurements (described in Section CEOAE Measurements). A 60 dB SPL CAS presentation level is commonly used for MOC reflex experiments, as it is the highest BBN level, on average, that elicits MOC reflex activity without triggering the middle ear muscle reflex (Guinan, 2006). CAP level-series were obtained using a chained stimulus paradigm (Hamill et al., 1991), which randomized stimulus levels from 50 to 80 dB ppeSPL using 10 dB steps. Each of the interleaved recording blocks automatically stopped after 2,048 averages were collected at each of the four stimulus levels and a 120 s break was inserted between each interleaved trial to allow subjects to reposition, etc. Advantages of using the chained paradigm in this context were that complete level-series functions could be obtained relatively quickly (~12 min) in a single testing block and that the effects of electrophysiologic or myogenic noise were randomly distributed across responses to all stimulus levels as opposed to one. In a typical recording session, three to four pairs of level-series functions without and with noise were obtained and averaged at the end of the session. Each recording session thus resulted in eight grand average waveforms (2 conditions × 4 stimulus levels) with each grand average waveform being comprised of ~6,144–8,192 sweeps. At the end of six recording sessions, there were 48 waveforms (2 conditions × 4 stimulus levels × 2 stimulus types × 3 sessions) for each subject.

The 48 CAP waveforms for each subject were saved as ASCII files and analyzed offline in MATLAB. CAP waveforms were grouped based on stimulus type (click or chirp), level (50–80 dB ppeSPL), and whether they were obtained without or with CAS. CAP amplitudes for each waveform were expressed in two different ways: (1) ***Raw amplitude*** was calculated as the μV difference between the pre-stimulus baseline average amplitude and the N1 peak, which was automatically selected as the largest waveform minimum within a restricted time epoch at each level based on normative click and chirp latency data from our laboratory. Responses were “not present” if the raw amplitude of a peak was less than one standard deviation of the pre-stimulus baseline amplitude. (2) ***Normalized amplitude*** expressed each CAP peak magnitude as a percentage of the maximum raw amplitude (either without or with CAS) in the level-series in which it was acquired:

$$\begin{array}{l} Normalized\;Amplitude=\\ \left(\frac{\text{Raw CAP Amplitude}\left(\text{uV}\right)}{\text{Single Session Level Series Maximum Amplitude }\left(\text{uV}\right)}\right)\times 100 \end{array}$$

Treating the data in this manner produced normalized level-series functions for each subject at the end of each recording session. We hypothesized two advantages to this approach. First, normalizing data obtained in each recording session would be expected to minimize differences in raw CAP amplitudes within subjects that were due to changes in electrode placement or orientation in the ear canal between visits, which can significantly influence raw amplitudes (e.g., Alhanada, 2012). Second, a normalized scale would be expected to make level-series functions between subjects more similar; because we analyzed group data in this experiment, it was imperative to reduce the effects of inter-subject differences in raw amplitude on our results.

#### CEOAE measurements

Three pairs of CEOAE level-series functions (60-80 ppeSPL^1^) without and with CAS were obtained using a Mimosa Acoustics HearID System (Mimosa Acoustics, Inc. Champaign, IL). Responses were collected using “linear” clicks (i.e., consistent stimulus polarity and level across all presentations) presented at 11/s for 250 sweeps in each trial. CEOAEs were considered present if they were ≥6 dB above the noise floor and if emission waveform sub-averages from response bins A and B were ≥80% correlated. CEOAE files were saved and offline analyzed in MATLAB, which extracted the composite values representing total emission amplitude and noise floors for each level and CAS condition. All response amplitudes were converted from dB to a pressure scale in order express CEOAE level-series on a linear ordinate scale, as was done with CAPs.

### Analyses

#### Chirp-to-click CAP amplitude ratios and amplitude comparisons

Chirp-to-click CAP amplitude ratios were calculated for each stimulus level using grand-averaged chirp and click *raw amplitudes* obtained without CAS for each subject. The purpose of this analysis was to determine the relative amplitude advantage of the chirp at each stimulus level. Paired *t*-test comparisons between chirp and click raw amplitudes without CAS at each level were also conducted.

#### CAP inhibition measurements: amplitude reduction and effective attenuation

The first step in testing the hypothesis that chirp-evoked CAPs were more sensitive to MOC reflex inhibition than clicks was to determine whether level-series functions were less variable when expressed either in units of *raw amplitude* or *normalized amplitude*. While we expected that normalizing amplitudes for each recording session would decrease between-subject CAP amplitude variability and provide a better scale on which to analyze group data, this was tested empirically. Coefficients of variation, which allow for variability comparisons between data sets with different units (e.g., μV vs. %), were calculated at each level and compared for raw and normalized level-series functions for each stimulus type. The amplitude scale producing the smallest coefficients of variation at each stimulus level and across all stimulus levels was used in subsequent analyses of CAP inhibition under the assumption that the less variable scale would be more sensitive to “true” physiologic changes induced by the MOC reflex.

Group CAP inhibition for chirps and clicks was quantified using two measures reported in the literature: (1) ***Amplitude reduction*** was calculated as the average “vertical” (ordinate) difference in CAP amplitudes without and with CAS at each level of the level-series function. This method of quantifying MOC reflex strength is most commonly used in the OAE inhibition literature; (2) ***Effective attenuation*** of chirp and click CAPs was calculated as the “horizontal” (abscissa) difference between linear regression fits to level-series without and with CAS using all subject data. Effective attenuation expresses the amount of dB that the *stimulus* would need to be increased to overcome the effects of MOC reflex inhibition; it is therefore useful in quantifying inhibition in terms of input level, which allows for gross comparisons of pre-neural and neural responses on the same scale (e.g., Puria et al., 1996).

## Results

### Amplitude differences between chirp- and click- evoked CAPs

With few exceptions, chirps produced larger raw peak amplitudes than clicks in individual ears, as evidenced by chirp-to-click CAP amplitude ratios (Figure 1). The size of the chirp/click amplitude ratio differed between subjects and showed a range of 0.76–4.22 across all stimulus levels. For most participants, the amplitude ratios decreased slightly as level was increased. Note that click-evoked responses at 50 dB ppeSPL were separable from the noise floor in all three test sessions in only 9 of the 14 participants; thus, amplitude ratios were calculated for only 9 participants at this level.

**Figure 2:**
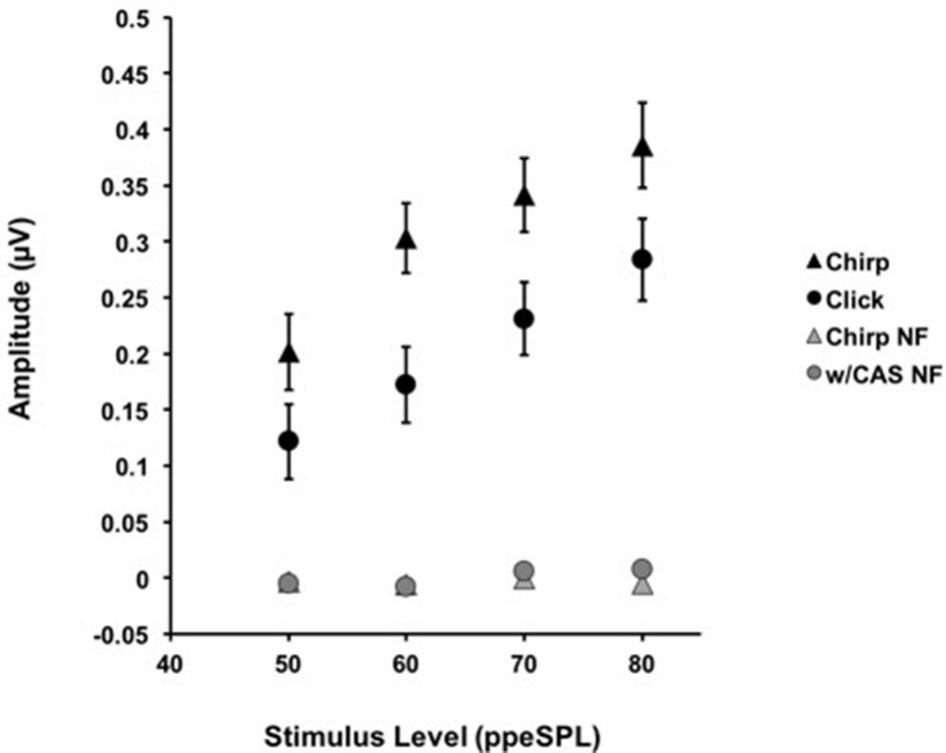
**The average amplitudes for CAPs in response to chirps (▲) and clicks (•) without CAS are shown as a function of level**. Chirp raw amplitudes were significantly larger than click raw amplitudes at every level using a corrected alpha level for multiple comparisons (α = 0.0125). Error bars = SEM; NF = Noise Floor.

The mean *raw amplitudes* of chirp-evoked CAPs without CAS were larger than those for clicks at each level tested (Figure 2). Paired *t*-tests with Bonferroni corrections for multiple comparisons (α = 0.0125) revealed that these differences were significant at 50 [*t*_(8)_ = −2.85, *p* = 0.008], 60 [*t*_(13)_ = −7.19, *p* = 0.0009], 70 [*t*_(13)_ = −4.28, *p* = 0.001], and 80 dB ppeSPL [*t*_(13)_ = −2.57, *p* = 0.007].

### CAP inhibition

**Figure 3:**
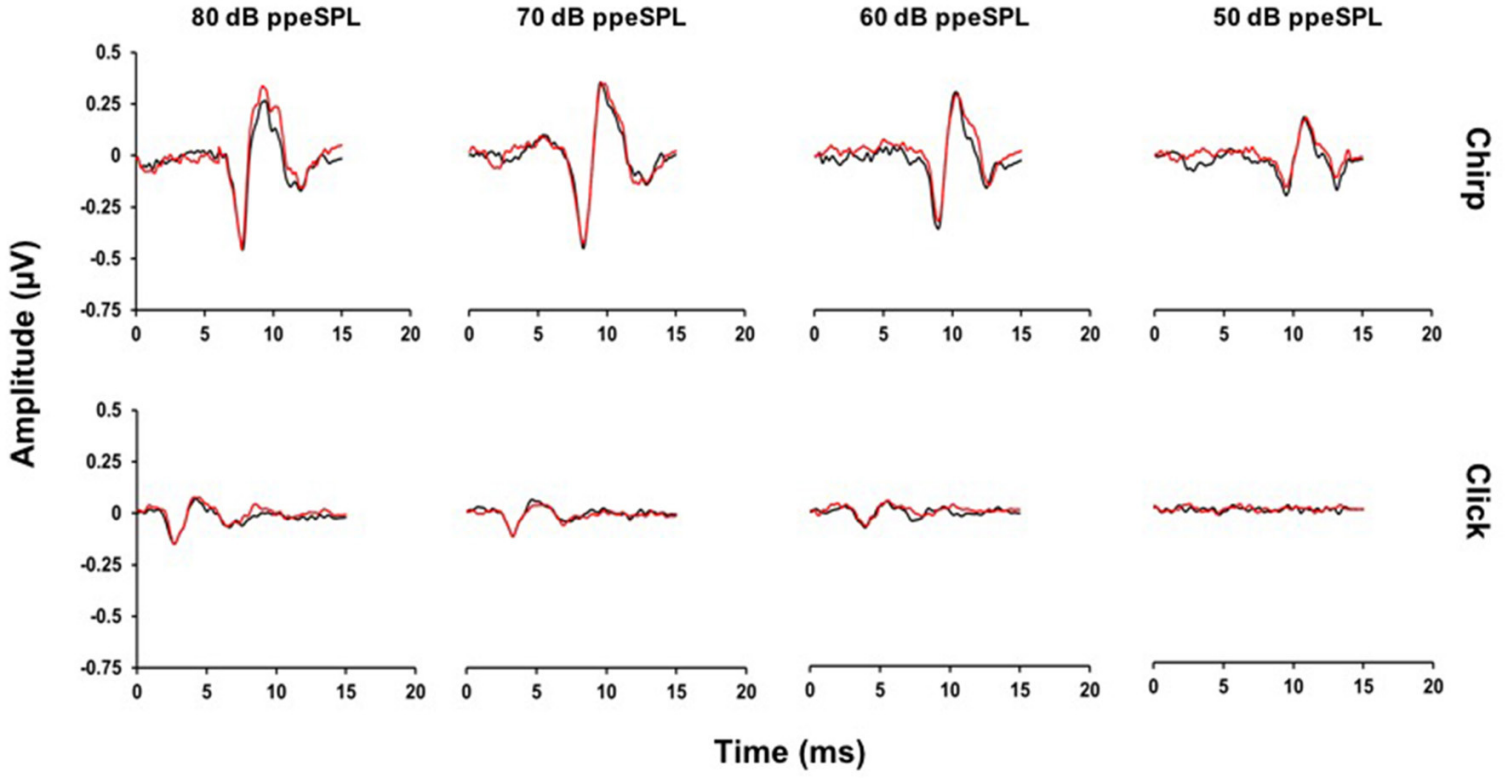
**Average CAP waveforms evoked by chirps (top)** and clicks **(bottom)** for a representative subject. Small reductions in N1 peak amplitudes with CAS can be seen in the chirp-evoked CAP waveforms at lower stimulus levels, but not for the click at any level. Note that at 50 dB ppeSPL, a click-evoked CAP was not identified in this subject (Black = without CAS; Red = with CAS).

Representative chirp- and click-evoked CAP waveforms without and with CAS from a randomly selected participant are plotted in Figure 3. This figure demonstrates three pertinent observations that were noted in most subjects including: (1) the overall amplitude advantage of chirps, especially at lower stimulus levels, (2) the small reductions in chirp- and click-evoked CAP amplitudes with CAS, and (3) the stability of pre-stimulus baselines prior to the N1 peak of the CAP.

**Figure 4:**
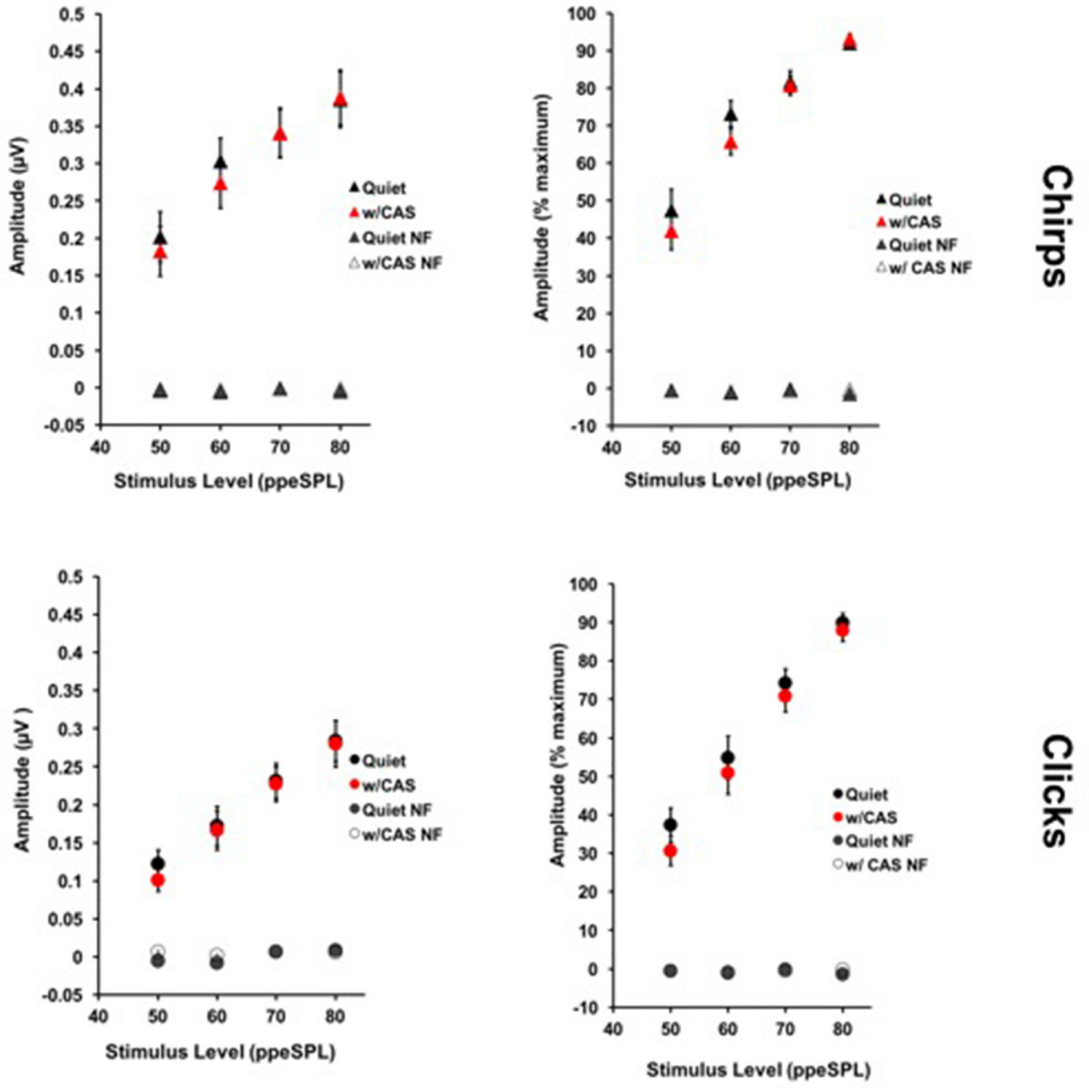
**Average level-series functions for chirps (top)** and clicks **(bottom)** expressed in raw **(left)** and normalized **(right)** amplitudes. Response variability was smaller across all subjects when amplitudes were expressed on a normalized scale. Error Bars = SEM; NF = Noise Floor.

Figure 4 displays chirp and click average level-series functions across all subjects and sessions without and with CAS. Level-series functions are expressed in both normalized and raw amplitudes for each stimulus type. For chirps, the average coefficient of variation across four stimulus levels and two noise conditions was 45% when expressed in raw amplitude and 20% when expressed in normalized amplitude; this mean difference was significant [*t*_(14)_ = 3.46, *p* = 0.0038]. For clicks, the average coefficient of variation was 47% when expressed in raw amplitude and 29% when expressed in normalized amplitude, which was also a significant mean difference [*t*_(14)_ = 2.86, *p* = 0.013]. Thus, we used the less-variable measurements expressed in *normalized amplitude* for subsequent MOC reflex inhibition of CAP analyses.

#### Normalized CAP amplitude reductions

**Figure 5:**
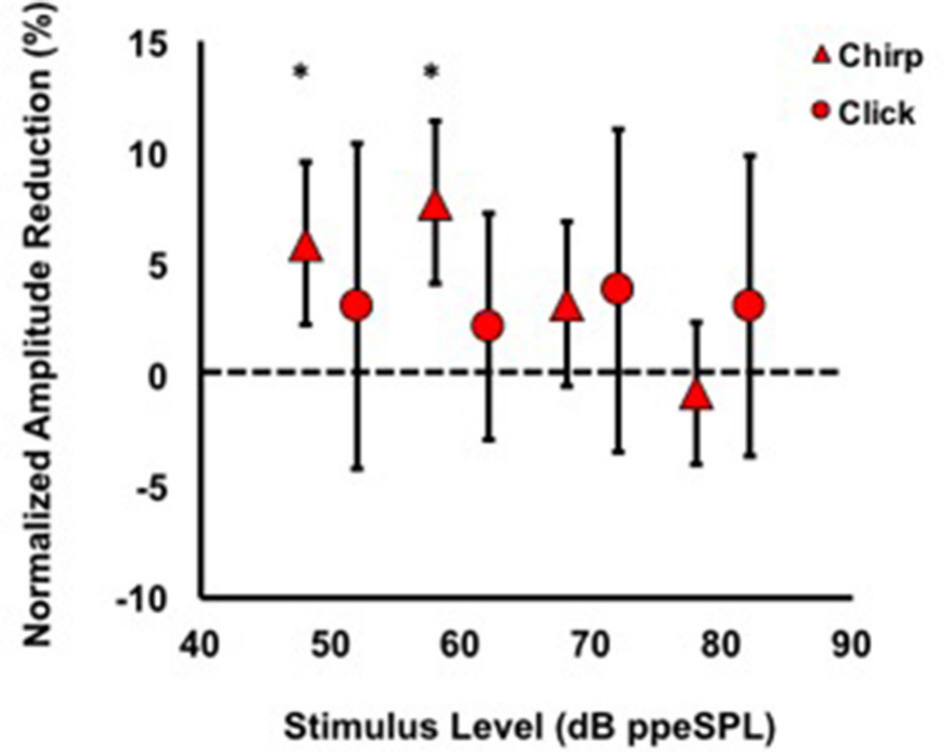
**Average normalized chirp and click amplitude reductions for all subjects**. Asterisks indicate significant reductions (*p* <0.01). Note that at every stimulus level, chirp inhibition was less variable than clicks, as indicated by the 95% confidence interval bars.

Average normalized amplitude inhibitions were largest for stimulus levels below 80 dB ppeSPL for both chirps and clicks (Figure 5). Normalized amplitude reduction with CAS was statistically significant only for chirp-evoked responses at 50 [*t*_(30)_ = 3.55, *p* = 0.0006] and 60 dB ppeSPL [*t*_(38)_ = 4.18, *p* < 0.0001], respectively, using an alpha level (α = 0.0125) to account for multiple comparisons.

#### CAP effective attenuation

**Figure 6:**
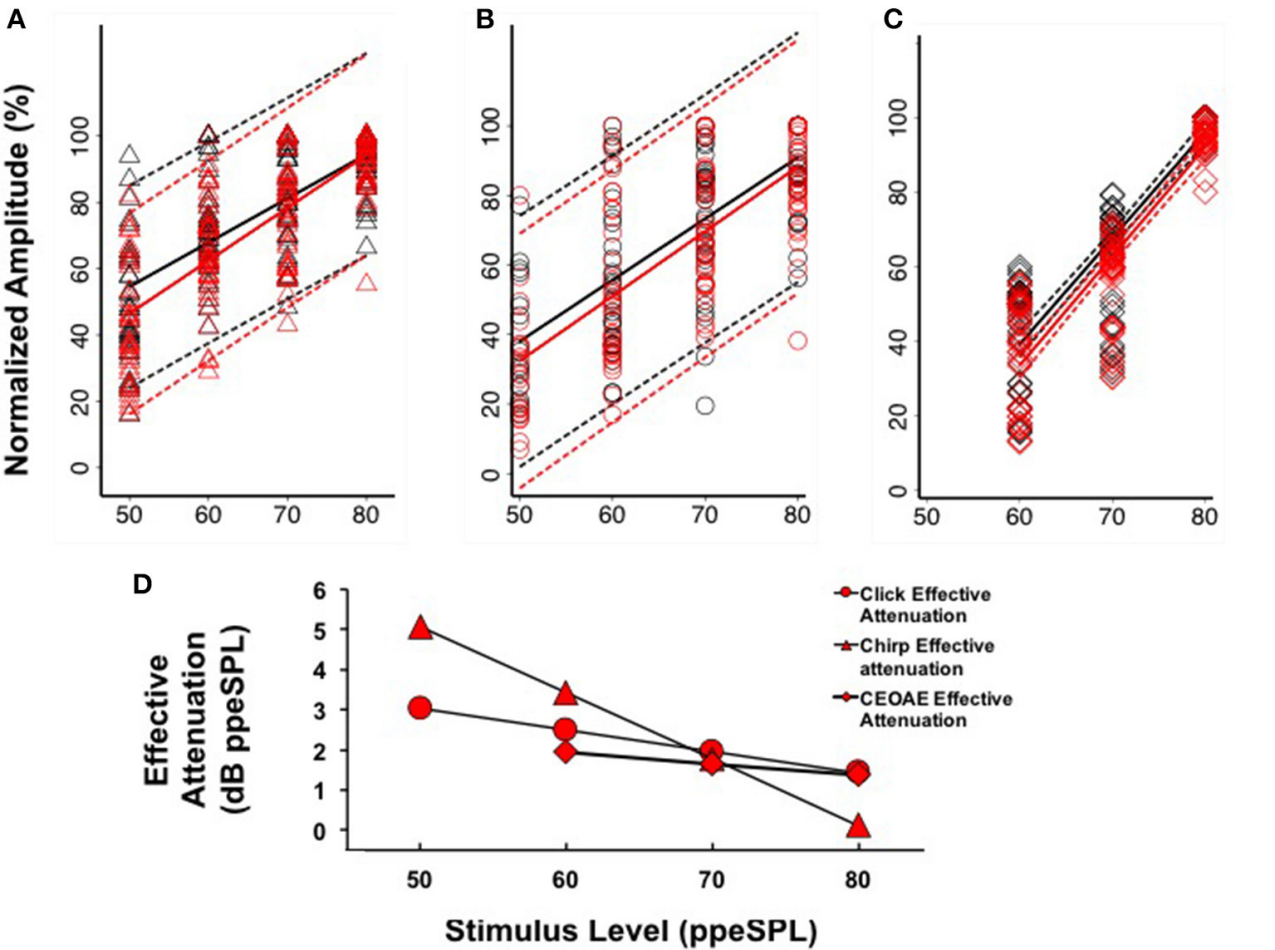
**Linear regression fits (with 95% CIs) to normalized chirp CAP (A)**, click CAP **(B)**, and CEOAE **(C)** level-series functions. Effective attenuation **(D)** was calculated as the abscissa (horizontal) difference between linear fits to without and with CAS group data.

Separate linear regression models were fit to the normalized group level-series data^2^ obtained without and with CAS for chirps (*y* = 1.33x–11.96, *R*^2^ = 0.47; *y* = 1.59x–33.22, *R*^2^ = 0.57) and clicks (*y* = 1.77x–50.13, *R*^2^ = 0.51; *y* = 1.86x–60.75, *R*^2^ = 0.54), respectively (Figures 6A,B). For both stimulus types, the models fit to CAP amplitudes without and with CAS diverged at low stimulus input levels and converged at higher stimulus input levels, indicating a greater effect of CAS on CAP amplitudes at low input levels. Regression coefficients as a function of condition (without or with CAS) were not significantly different for chirps (*t* = 1.63, *p* = 0.103) or clicks (*t* = 0.45, *p* = 0.66). Effective attenuation for each stimulus type was calculated as the difference in the abscissa between without and with CAS linear regression lines for equivalent ordinate values (Figure 6D). At the lowest stimulus level, effective attenuation was 5.07 dB for chirps and 3.02 dB for clicks (Figure 6D).

#### Comparison of CAP and CEOAE effective attenuation

Based on our findings that CAP amplitudes were less variable when expressed on a normalized scale (see Section CAP Inhibition), we only report CEOAE inhibition in terms of effective attenuation of normalized responses in the present experiment for comparison. CEOAE normalized level-series data from all subjects obtained without (*y* = 2.84x–131.42, *R*^2^ = 0.78) and with CAS (*y* = 2.92x–141.87, *R*^2^ = 0.80) were also fit with separate linear regression models (Figure 6C). The CEOAE models were better fit than CAP data, as normalized CEOAE amplitudes were less variable across subjects. The largest differences in without and with CAS models occurred at the lowest input level, as was observed in the CAP data. Regression coefficients as a function of condition (without or with CAS) were not significantly different (*t* = 0.43, *p* = 0.67). Effective attenuation was calculated in the same manner as the CAP data. A comparison of chirp-evoked CAP, click-evoked CAP, and CEOAE effective attenuations at 60 dB ppeSPL revealed that inhibition was largest for chirps (3.42 dB), followed by click CAPs (2.49 dB) and CEOAEs (1.93 dB).

## General discussion

The findings of this study were that: (1) Chirps evoked larger CAP amplitudes than clicks at low to moderate stimulus levels; (2) Normalized CAP amplitude reductions with CAS were largest at the group level using chirps at 50 and 60 dB ppeSPL (5.89 and 7.75%, respectively). These were the only statistically significant amplitude reductions observed; (3) Effective attenuation measurements were largest at the group level for chirp-evoked CAPs followed by click-evoked CAPs and CEOAEs, respectively, at the lowest stimulus levels where all three could be measured (i.e., 60 dB ppeSPL).

### The chirp advantage

The chirp generated larger CAP amplitudes at each stimulus level in most subjects; however, the size of this advantage varied considerably across subjects. This finding is consistent with the observations of Chertoff et al. (2010; see Figure 3) who used higher presentation levels than the present study. Inter-subject differences in the chirp advantage may be related to multiple factors. First, the chirp used in the present study and by Chertoff et al. (2010) related frequency to basilar membrane delay using derived band CAP latencies from 15 normally hearing subjects reported by Eggermont (1979). Subject characteristics, such as sex, were not reported in that study, but it has been inferred that sex differences in cochlear length may affect basilar membrane delays and therefore the degree to which neural responses are synchronized (e.g., Don et al., 1993, 1994). With the current participant pool of 10 females and 4 males, it is possible that the chirp was not optimized for individual ears based on these differences. One way to quickly construct a CAP chirp that is more optimized for an individual ear than a click may be to use basilar membrane delay estimates from OAEs, as derived-band CAP masking procedures are time consuming. Secondly, some authors have encouraged the use of chirps that are optimized for different presentation levels (Elberling and Don, 2010; Elberling et al., 2010; Kristensen and Elberling, 2012), suggesting that cochlear frequency place maps do not scale simply with level. Our use of a chained stimulus paradigm, which allows for random level presentation of a *single* stimulus file, did not provide the flexibility to use multiple chirps optimized for different levels in this investigation.

The chirp advantage reported here and by Chertoff et al. (2010) suggests that chirps may also be a useful tool in studying animal and human synaptopathy—a pathology in which noise exposure predominately insults high threshold auditory nerve fibers but spares low threshold fibers and hair cells (Kujawa and Liberman, 2009). Synaptopathy has been postulated as the basis of severe hearing difficulties in patients with normal audiograms (i.e., “hidden hearing loss”) and may also be involved in the generation of tinnitus (e.g., Schaette and McAlpine, 2011). The synaptopathy “phenotype” in animal models presents as significantly reduced CAP amplitudes evoked by suprathreshold sounds in the presence of normal (electrophysiologic) audiometric thresholds and OAE responses. Because chirp-evoked CAPs are larger and represent the summed activity from auditory nerve fibers along the length of the basilar membrane, they may provide a more sensitive measure of synaptopathy. Further, narrowband chirps tailored to evoke CAPs may be even more sensitive to synaptopathy in distinct cochlear regions.

### MOC reflex effects on CAPs

Our findings suggest that chirps may be more suitable than clicks in studying the neural consequences of MOC reflex inhibition for a few reasons. First, chirp-evoked CAPs were larger than clicks even at the lowest stimulus level, which allowed for more accurate N1 peak identification in quiet and with CAS conditions (e.g., compare 60 dB ppeSPL waveforms for chirps and clicks in Figure 3). Since OHCs are more potently inhibited by the MOC reflex at low input levels, using a chirp may allow for more accurate estimates of MOC effects in this range. Because chirp-evoked CAPs reflect the summed activity over broader cochlear regions, they may also be more sensitive to the summated effects of MOC fibers than click-evoked CAPs, which mainly reflect neural synchrony from fibers innervating the cochlear base (Don and Eggermont, 1978). Second, the variability of CAP inhibition for chirps was smaller relative to clicks on both amplitude reduction and effective attenuation measurement scales (Figures 5, 6A,B). This finding suggests that chirps may be more sensitive to “true” physiologic changes attributable to MOC reflex activation than clicks. It is important to note, however, that chirp and click CAP effective attenuation was calculated from relatively weak linear regression fits to *group* data, which may have been caused by individual differences in both level-series function contours and magnitude of inhibition. An analysis of individual data using the same method resulted in even poorer linear fits due to the fewer data points in the models. Thus, a limitation of our work is that we were unable to reliably resolve efferent inhibition of CAPs at the single-subject level, which is of interest in studying individual variation in MOC function and in understanding the predictive relationships between pre-neural and neural efferent assays. This issue may have been resolved by focusing recording time on obtaining more response averages to fewer low-intensity input levels (Lichtenhan et al., 2016); however, an advantage of acquiring a level-series function spanning 40 dB was that CAS effects on CAPs evoked by different stimulus levels could be evaluated.

Involvement of the middle ear muscle reflex is always a consideration in MOC reflex experiments, as CAS can activate both mechanisms. The observed CAP amplitude reductions with CAS were unlikely to be the consequence of “sub-threshold” middle ear compliance changes from activation of the middle ear acoustic reflex because such a change would be expected to reduce responses to *all* input levels of the level-series function. In contrast, the CAS-induced changes in our data were primarily at low input levels, which is suggestive of changes in OHC function. Nevertheless, the possibility of middle ear muscle involvement cannot be fully ruled out, as some reports indicate that standard measures of acoustic reflex threshold, like the one used in our screening protocol, may overestimate the level at which the stapedius muscle is activated by CAS (Feeney and Keefe, 2001; Zhao and Dhar, 2010).

### CEOAE and CAP effective attenuation comparisons

OAE measurements are used far more often as an indirect assay of MOC reflex effects than CAP amplitudes, as they require less time to collect and are less inherently variable than electrophysiologic techniques (see Figure 6). This difference is presumably because far-field CAP recordings are influenced by more sources of noise (e.g., background EEG, myogenic and electrical noise, high electrode impedance due to small surface area) than OAEs. Based on these technical differences, a compelling argument can be made for using OAE based assays of the MOC reflex in a clinical setting, for example. It is, however, of great importance to understand the relationships between pre-neural and neural inhibition because the latter reflects modulation of the auditory nerve signal mediating hearing, which cannot be assessed with OAEs. Pre-neural and neural inhibition comparisons must be made in light of evidence that there is not a one-to-one correspondence between changes in OHC function and modulation of IHC neurotransmitter release, which is the basis for auditory nerve fiber depolarization (Guinan, 2012). However, by expressing CEOAE and CAP inhibition in terms of effective attenuation, direct comparisons can be made between MOC reflex effects on each type of response.

Our observation that CEOAE effective attenuation *underestimated* chirp- and click-evoked CAP effective attenuation by up to ~1.5 dB at low stimulus levels was consistent with previous reports in animals and humans (e.g., Puria et al., 1996; Lichtenhan et al., 2016). The source of this consistently reported discrepancy is not clear. OHCs are postsynaptic only to MOC fibers, whereas the auditory nerve is postsynaptic to both MOC and lateral olivocochlear (LOC) fibers, which directly contact type I auditory nerve fibers (Warr and Guinan, 1979). This anatomical configuration suggests that CAP inhibition reflects the summation of MOC and LOC inhibition, whereas OAEs only reflect MOC inhibition. However, several lines of evidence appear to refute this suggestion. Gifford and Guinan (1987) measured CAP inhibition from cats while electrically stimulating different regions of the caudal brainstem. They observed that stimulating the floor of the fourth ventricle (which diffusely activates the OCB proper) is comparable to the combined inhibitory effects of directly stimulating MOC neurons. When LOC neurons were directly stimulated, no inhibitory effects on the CAP were observed. The investigators also documented that increases in cochlear microphonic amplitude were related to decreases in CAP amplitude during OCB stimulation, indicating that the same process (i.e., direct modulation of OHCs) likely mediates each effect. Brown et al. (1983) measured IHC receptor potential tuning curves (from the AC component) with and without fourth ventricle electrical stimulation and observed 9–24 dB of inhibition at the tuning curve “tips” (i.e., center frequency) with no change away from center frequencies. Basilar membrane displacement tuning curves show similar effects (Murugasu and Russell, 1996; Cooper and Guinan, 2003). While these measurements are pre-neural, they are remarkably similar to auditory nerve tuning curves using the same paradigm (Wiederhold and Kiang, 1970; Bonfils et al., 1986). Thus, there is strong evidence that the MOC system is the main effector of inhibition in both *pre-neural* and *neural* assays. In contrast, there is no evidence that the LOC system can be excited with acoustic stimulation and its role in hearing remains poorly understood. The best available evidence suggests that the LOC system's influence on hearing is likely through slow “top-down” potentiation of auditory nerve activity (Sahley and Nodar, 1994; Groff and Liberman, 2003; Le Prell et al., 2005), which may protect auditory nerve fibers from acoustic trauma (e.g., Darrow et al., 2007).

If the MOC reflex accounts for inhibition measured from both OHCs and the auditory nerve, it may be expected that effective attenuation slopes of CEOAEs and CAPs would be parallel. We observed at the group level that the slopes of click CAP and CEOAE effective attenuation were similar to each other and quite different than chirp CAP effective attenuation (Figure 6D). Because we did not measure chirp-evoked OAEs, it is unclear if this difference is stimulus related or explained by some other mechanism. The temporal differences between clicks and chirps make the chirp a better stimulus for evoking synchronized *neural* responses, but these differences would not be expected to produce significantly dissimilar composite emission amplitudes evoked by each stimulus. Previous work has indicated that stimulus frequency OAEs (SFOAEs) and CEOAEs are generated in a nearly equivalent manner through coherent reflection when the spectral power within a bandwidth on the basilar membrane is equal (Neumann et al., 1994; Kalluri and Shera, 2007); if a chirp is conceptualized as a swept SFOAE, the effect of MOC reflex inhibition on chirp-evoked OAEs and CEOAEs would be expected to be similar. To our knowledge, there have been no experiments comparing MOC reflex inhibition of click- and chirp-evoked OAEs; therefore, the origin of the differences in effective attenuation slopes between chirp-evoked CAPs and pre-neural and neural measurements evoked with clicks in the group data is not clear.

## Conclusions

The present study is the first in which a chirp was used to evoke CAPs from the human auditory nerve with and without MOC reflex activation. Our findings indicate that, at least at the group level, the chirp may be a more sensitive stimulus for evaluating *neural* efferent effects than a click because it evokes a larger response at lower stimulus intensities and may be more sensitive to summed efferent activity along the cochlear spiral. Additionally, our findings are consistent with previous work indicating that OAE assays of the MOC reflex underestimate neural inhibition (i.e., Puria et al., 1996; Lichtenhan et al., 2016). Future experiments which optimize chirp parameters for individual ears and allow for reliable within-subject neural measurements of MOC reflex inhibition are warranted.

## Ethics statement

This study was carried out in accordance with the recommendations of the Arizona Human Protections Program with written informed consent from all subjects.

## Author contributions

SS developed the study, collected data, and ran analyses. JL supplied the script for generating the chirp stimulus and contributed to the theoretical development of the study. BC was also instrumental in study design and data analysis.

## Funding

Financial disclosures: This research was funded by the National Institutes of Health, National Institute on Deafness and other Communication Disorders (F30 DC01418 and R01 DC014997).

### Conflict of interest statement

The authors declare that the research was conducted in the absence of any commercial or financial relationships that could be construed as a potential conflict of interest.

## Footnotes

^1^CEOAE responses to clicks at 50 dB ppeSPL were absent in most subjects based on our criteria; therefore, CEOAE level-series measurements were made from 60 to 80 dB ppeSPL.

^2^Note that while normalizing CAP amplitudes to the maximum value in a subject's level-series function reduced amplitude variation across all levels, it also introduced heteroscedasticity; therefore, robust standard errors were used for each regression model, which allowed for the presence of heteroskedastic data by relaxing the assumptions that errors were independent and identically distributed (Hayes and Cai, 2007).
